# The saliva microbiome profiles are minimally affected by collection method or DNA extraction protocols

**DOI:** 10.1038/s41598-017-07885-3

**Published:** 2017-08-17

**Authors:** Yenkai Lim, Makrina Totsika, Mark Morrison, Chamindie Punyadeera

**Affiliations:** 1 The School of Biomedical Sciences, Institute of Health and Biomedical Innovation, Queensland University of Technology, 60 Musk Avenue, Kelvin Grove, Brisbane, QLD 4059 Australia; 2 Translational Research Institute, Woolloongabba, Brisbane, QLD 4102 Australia; 3 The University of Queensland Diamantina Institute, The University of Queensland, Translational Research Institute, Woolloongabba, Brisbane, QLD 4102 Australia

## Abstract

Saliva has attracted attention as a diagnostic fluid due to the association of oral microbiota with systemic diseases. However, the lack of standardised methods for saliva collection has led to the slow uptake of saliva in microbiome research. The aim of this study was to systematically evaluate the potential effects on salivary microbiome profiles using different methods of saliva collection, storage and gDNA extraction. Three types of saliva fractions were collected from healthy individuals with or without the gDNA stabilising buffer. Subsequently, three types of gDNA extraction methods were evaluated to determine the gDNA extraction efficiencies from saliva samples. The purity of total bacterial gDNA was evaluated using the ratio of human *β-globin* to bacterial 16S rRNA PCR while 16S rRNA gene amplicon sequencing was carried out to identify the bacterial profiles present in these samples. The quantity and quality of extracted gDNA were similar among all three gDNA extraction methods and there were no statistically significant differences in the bacterial profiles among different saliva fractions at the genus-level of taxonomic classification. In conclusion, saliva sampling, processing and gDNA preparation do not have major influence on microbiome profiles.

## Introduction

As a biospecimen, saliva is less utilised in a Clinical Chemistry Laboratory compared to tissue, blood, urine and faecal matter despite being the most easily accessible non-invasive body fluid^1^. This is in part due to the lack of standardised saliva sample collection protocols and our limited knowledge about the diurnal variability of biomolecules in saliva^2^. Unlike other biospecimens, saliva sample types (whole-mouth unstimulated saliva, acid and mechanically stimulated saliva, oral swab and oral rinse) may differ in their composition and may have an impact on the analytes to be detected^3^. In addition, the relatively low abundance of biomolecules in saliva makes it more challenging albeit using advanced technologies^4–7^. To date, salivary biomarkers of potential diagnostic value has been identified and validated for both oral and systemic diseases^8–15^. Currently, salivary DNA based methods are used in many diagnostic laboratories for mutations and polymorphisms studies relating to cancers and hereditary disorders^16^.

The microbial communities resident at different sites of the human body are widely recognised for their roles in protecting, initiating, and facilitating disease pathogenesis, and the oral cavity is relatively understudied in this regard. Reliable characterisation of these microbial communities in various disease states should also support the development of new saliva-based diagnostics and therapeutics^17–20^. Historically, oral microbiota research has heavily depended on microbial specific cultures although many researchers adapted to cultivation-independent molecular techniques to more holistically assess microbiome (the collective genomes of microorganisms) changes^21, 22^. The advent of high-throughput genomic (g)DNA sequencing methods has revolutionized the field of human microbiome research, with particular focus to date on the gut microbiome using faecal samples.

In 2012, the International Human Microbiome Standards (IHMS) were concerned that the sample collection, processing and gDNA preparation of faecal samples may influence the data generated in human metagenomics research studies. Supported by the European Commission, a suite of sample collection and processing procedures were tested and gDNA preparation was carried out by IHMS contributors across 12 different countries. Contributors were asked to extract gDNA from the provided faecal samples using their own laboratory procedures as well as other standard protocols from literatures. After a thorough investigation such as the gDNA yield, quality and recovery of diversity and specific bacterial taxa, a set of 14 standard operating procedures were designed to help optimise data quality and comparability in the human microbiome field (http://www.microbiome-standards.org). As the next-stage forward, this study will investigate the influence of sample collection, processing and gDNA preparation with regards to saliva samples.

Aims of this study were three-fold: (i) to investigate the influence of saliva collection method on microbial analysis; (ii) to evaluate the most efficient bacterial gDNA extraction protocol from human saliva and; (iii) to determine the best saliva fraction for oral microbial studies. While different saliva collection methods are known to influence the composition of saliva biomolecules, we demonstrate that it does not contribute significantly to the oral microbiome profile. In addition, the overall bacterial gDNA yield was not affected by different extraction protocols when repeated bead-beating with lysis-buffer was implemented. However, the Maxwell® 16 LEV blood DNA kit was able to significantly increased the purity of the bacterial gDNA.

## Methods and Materials

### Study cohort and sample collection

This study was approved by the Queensland University of Technology (HREC no.: 1400000617) Medical Ethical Institutional Board and informed consent was obtained from all participants. All methods in this study were performed in accordance with the relevant guidelines and regulations. We have recruited normal healthy controls (n = 40) from the general population based on stringent recruitment criteria (Supplementary Data 1) to minimise baseline variations that may potentially affect the experimental endpoints. All volunteers (between 20 to 30 years of age) were self-reported to be in good general health with no underlying diseases, not receiving local and/or systemic antibiotics and no history of smoking and drinking habits.

**Figure 1:**
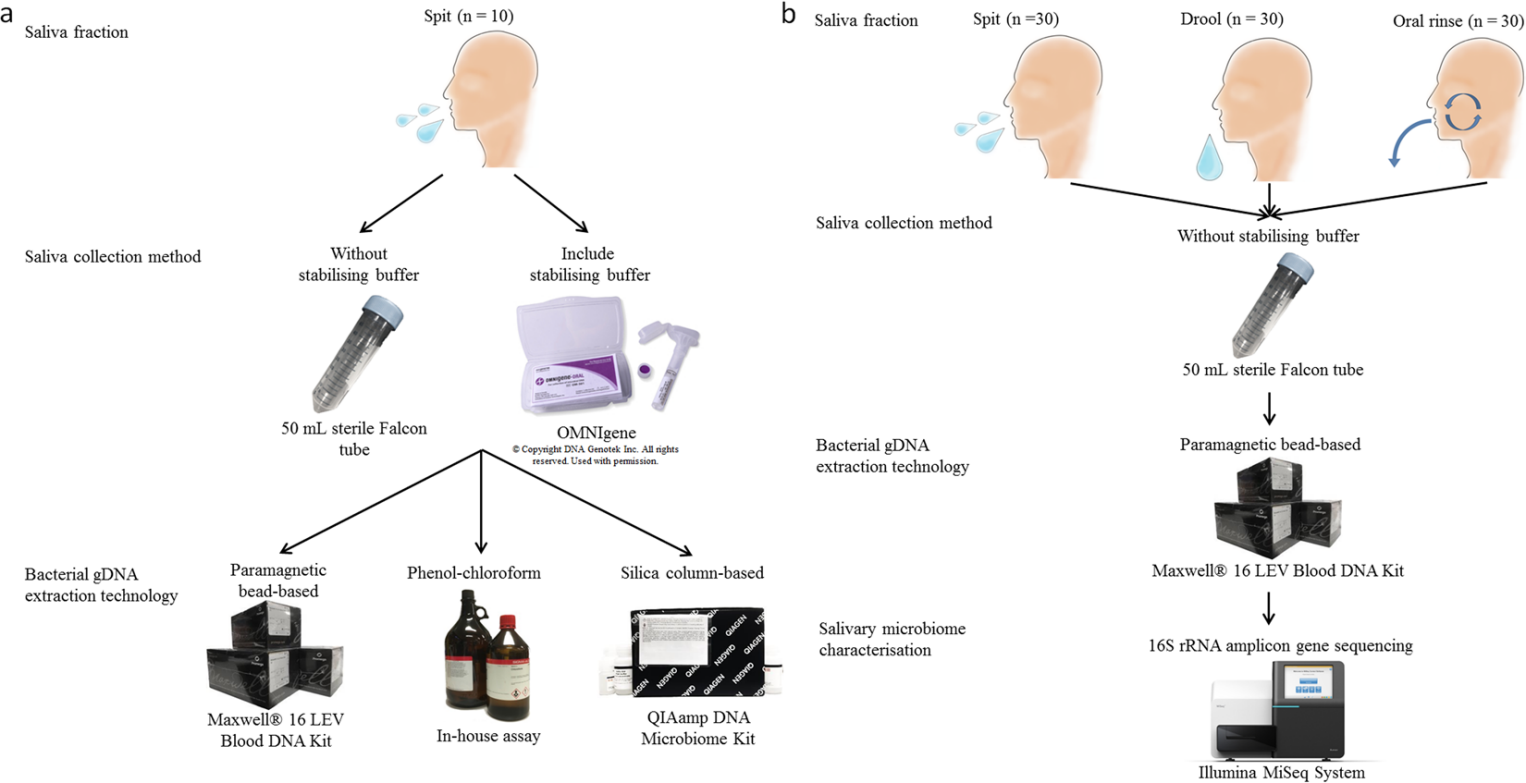
Study design overview. From Fig. 1a, Maxwell® 16 LEV Blood Kit was found to be the most efficient bacterial gDNA extraction method when spit samples were collected in 50 mL sterile Falcon tube. Hence, OMNIgene and other salivary bacterial gDNA extraction methods were excluded from the second part of the study (Fig. 1b).

Volunteers were asked to refrain from eating and drinking for an hour prior to donating saliva samples as per our previous work^9, 10, 12^. The volunteers were asked to sit in a comfortable position and were asked to rinse their mouths with bottled water to remove food debris. A set of two spit saliva samples were collected from ten volunteers (total samples, n = 20) in a 50 mL sterile Falcon tube (Becton, Dickinson and Company, New Jersey, USA) and an OMNIgene tube (DNA Genotek Incorporation, Ontario, Canada), respectively. Spit samples were collected by asking volunteers to forcefully spit saliva (not sputum) directly into the saliva collection devices (OMNIgene includes a separate mouth-piece that can be connected to the devise for saliva spitting and drooling proposes) (Fig. 1a). OMNIgene contained 1 mL of stabilising buffer, hence the maximum saliva sample that can be collected is 1 mL (OMNIgene tube capacity is 2 mL). Since 200 μL of saliva sample is required per extraction, a 1:1 mixture of spit sample collected from 50 mL sterile Falcon tube and 1 x phosphate-buffered saline (PBS) was used to have similar saliva sample volumes for microbial analysis.

Similarly three sets of saliva fractions were collected from a different cohort of 30 normal healthy volunteers (total samples, n = 90) in the order of spit, drool and oral rinse with five minute intervals. Drool samples were collected by asking volunteers to pool saliva in the mouth and expectorate naturally into a 50 mL sterile Falcon tube while spit samples were collected as described above. Oral rinse samples were collected by asking volunteers to swish and gargle with 10 mL of 0.9% (w/v) saline solution (Baxter International Incorporate, Illinois, USA) for a minute and expectorate into a 50 mL sterile Falcon tube as previously published (Fig. 1b)^8^. We have also tested the reverse order of sample collection to ensure that the order of collection does not influence the microbial diversity.

After collection, all samples were transported back to the laboratory on dry ice. Spit and drool samples were aliquoted evenly into 1.5 mL Eppendorf tubes (Eppendorf, Hamburg, Germany) and stored at −80 °C. Oral rinse samples were further processed by centrifugation at 1000 × g for 15 minutes at 4 °C to separate the cellular pellet from cell-free salivary supernatant. Cellular pellets were resuspended in sterile 1 x PBS and aliquoted evenly into 1.5 mL Eppendorf tubes and stored at −80 °C.

### Maxwell® 16 LEV blood DNA kit

Saliva samples (200 μL irrespective of the fraction) were subjected to centrifugation at 16000 × g for 10 minutes at 4 °C (to reduce bacterial activities in saliva samples) to separate the cellular pellet from cell-free supernatant. A volume of 500 μL of in-house lysis buffer (200 mL of 0.5 M sodium chloride, 0.05 M trisaminomethane at pH8, 0.05 M ethylenediaminetetraacetic acid at pH 8 and 4% sodium dodecyl sulfate) was added to the cellular pellet and mixed well by vortexing. The samples were then transferred to a 2 mL conical screw cap microtube with zirconimum beads (combination of 0.3 g of 0.1 mm and 0.1 g of 0.5 mm, Daintree Scientific, St. Helens, Tasmania, Australia) and the cells were disrupted using the Precellys® 24 (Bertin Corporation, Rockville, USA) at 5000 × g for 3 × 60 seconds. The homogenates were then incubated within a 70 °C water-bath for 15 minutes with the contents mixed at five minute intervals by gentle inversion of the tubes. After incubation, samples were centrifuged at 15000 × g for five minutes and the supernatant fractions were transferred to sterile 1.5 mL Eppendorf tubes containing 30 μL of proteinase-K (Maxwell® 16 LEV blood DNA kit), vortexed for 30 seconds, then incubated at 56 °C for 20 minutes. Total nucleic acids were then recovered from these samples using the blood DNA columns using the Maxwell® 16 MDx Research Instrument (Promega Corporation, Wisconsin, USA). Finally, 2 μL of 10 μg RNase A (Qiagen, Hilden, Germany) was added to the samples and incubated in a 37 °C water-bath for 15 minutes for RNA digestion.

### Phenol-chloroform method

The sample preparation and bacterial cell lysis for the phenol-chloroform extraction method was similar to the Maxwell® 16 LEV blood DNA method described above, with the following modifications. After the second incubation in a 56 °C water-bath for 20 minutes, equal volumes of buffer saturated phenol (Sigma Co., Victoria, Australia) was added and mixed by inverting several times and centrifuged at 18000 × g for 5 minutes at 4 °C. The aqueous layer was carefully removed into a new 1.5 mL Eppendorf tube without disturbing the interphase layer. An equal volume of chloroform:isoamyl (24:1) alcohol (Sigma Co., Victoria, Australia) mixture was added to the sample and vortexed for 5 minutes for further purification. Mixed samples were centrifuged at 18000 × g for 10 minutes at 4 °C and the aqueous layer was transferred into a new 1.5 mL Eppendorf tube. Depending on the volume of the aqueous layer, 1:10 of 3 M sodium acetate (pH 5.2) and an equal volume of 100% isopropanol were added to the sample and mixed well before incubating at −20 °C overnight. The samples were then centrifuged at 18000 × g for 10 minutes at 4 °C, the supernatant discarded and 500 μL of 70% ethanol was added to the pellet before centrifugation again at 18000 × g for 5 minutes at 4 °C. The supernatant was discarded and the gDNA samples were dried using a SpeedVac Concentrator SAVANT ISS110 (ThermoFisher Scientific, Massachusetts, USA) for 5 minutes at 60 °C. The gDNA was resuspended in 20 μL of 1 x trisaminomethane and ethylenediaminetetraacetic acid buffer and vortexed for a minute to mix well. Finally, 2 μL of 10 μg RNase A was added to the sample and incubated in a 37 °C water-bath for 15 minutes for RNA digestion.

### QIAamp DNA Microbiome Kit

The sample preparation and bacterial cell lysis for the QIAamp DNA Microbiome Kit (Qiagen, Hilden, Germany) extraction method was similar to the Maxwell® 16 LEV blood DNA method. However, the proteinase K from QIAamp DNA microbiome kit was used for the second incubation. The QIAamp DNA microbiome kit extraction was carried out according to the manufacturers’ instruction and gDNA was eluted in a final volume of 50 μL. A volume of 2 μL of 10 μg RNase A was added to the gDNA sample and incubated in a 37 °C water-bath for 15 minutes for RNA digestion.

### gDNA quantification

The quantity and quality of the extracted gDNA were evaluated using the Nano Drop ND-1000 spectrophotometer (ThermoFisher Scientific, Massachusetts, USA).

### Quantitative PCR

Bacterial 16S rRNA primer set (1114F-5′ CGGCAACGAGCGCAACCC 3′ and 1221R-5′ CCATTGTAGCACGTGTGTAGCC 3′) and human *β-globin* primer set (F-5′ CAACTTCATCCACGTTCACC 3′ and R-5′ GAAGAGCCAAGGACAGGTAC 3′) were used in quantitative (q)PCR reactions to determine the ratio of bacteria to human gDNA from the extracted samples. The reaction consisted of 5 μL of 2 × iTaq™ Universal SYBR® Green Supermix (Bio-Rad Laboratories, California, USA), 200 nM of forward and reverse primers for each of the respective genes and 20 ng of gDNA template. The total reaction volume (10 μL) was subjected to qPCR amplification using the conditions of an initial denaturing stage at 95 °C for 10 minutes and followed by 30 cycles of a minute at 60 °C. *Escherichia coli* gDNA was used as a positive control while DNase/RNase water was used as a negative control for the qPCR assay.

### 16S rRNA gene amplicon library preparation and sequencing

16S rRNA gene amplicons for sequencing by Illumina MiSeq system (Illumina Incorporate, California, USA) was prepared according to the manufacturers’ method with gene-specific sequences targeting the V6 and V8 hypervariable regions of the 16S rRNA gene^23^. Q5® Hot Start High-Fidelity (New England Biolads, Masachusetts, USA) polymerase enzyme was used for the index PCR instead of the recommended KAPA HiFi HotStart ReadyMix (Kapa Biosystem, Massachusetts, USA) polymerase enzyme, as the former enzyme yielded superior amplification efficiency with a lower error rate. Sequencing was performed at the Australian Centre for Ecogenomics (ACE, Brisbane, Australia).

### Statistical analysis

The statistical analysis for the investigation of the influence of saliva collection devices and the efficiency of saliva gDNA extraction methods were carried out by using GraphPad Prism (GraphPad Software Incorporate, California, USA). Since the quantity and quality of the extracted gDNA were not normally distributed, the non-parametric Wilcoxon matched-pairs signed rank test was used when comparing two different variables while the Friedman test was used when comparing more than two different variables. In addition, the Friedman test was also used to analyse the Ct mean variation of the bacterial 16S rRNA gene and the human *β-globin* gene of the gDNA extracted.

Illumina sequenced datasets were analysed using Quantitative Insights Into Microbial Ecology (QIIME) version 1.9.1 [PMID: 20383131] on the Ubuntu Linux virtual machine (Canonical Limited, London, United Kingdom). USEARCH 6.1 [PMID: 20709691] was used to perform a reference based chimera detection and removal to avoid perceived diversity^24^. Briefly, sequences were clustered into operational taxonomic units (OTUs) by PyNAST [PMID: 19914921] with a 97% sequence identity threshold against Greengenes core set database version 13.8 [PMID:16820507]^25^. An OTU table was generated with a list of prokaryotes and their respective observed OTU counts (abundance) for each sample based on the sequenced data in a biological observation matrix format. The OTU table was further edited to remove low abundance OTUs (≤0.1% of total sequences) and any sequences that were not of bacterial or archaeal origin. A subsampled OTU table was created by random sampling (without replacement) of the original OTU table based on the sample with the lowest OTU counts to account for different sequencing depths that may occur for each individual sample. The subsampled OTU table was used to calculate the α- (within-sample) and β- (between-sample) diversity metrics and to generate taxa summaries for each saliva fraction, from the phylum to genus levels of classification. Shannon index was used as an estimator for richness and evenness of the microbial community. Distance between samples was represented by the weighted (quantitative) and unweighted (qualitative) UniFrac distance metrics [PMID: 16332807] which are presented via principal component analysis (PCoA) plotting. Calypso version 5.4 (http://cgenome.net:8080/html/wiki/index.php/Calypso) was used to further analyse these data by providing statistical information on the significance of distribution for each genus as well as the combined genera in the three saliva fractions. Since the distribution of the genera between each saliva fraction is non-parametric, the Kruskal-Wallis rank test was used on Calypso to identify any significant differences in genera distribution. According to the p-values generated, there are no significant differences in the distribution of genera in spit, drool and oral rinse (Supplementary Data 6).

## Results

### The quantity and quality of total gDNA extracted using different saliva collection and extraction methods

**Figure 2:**
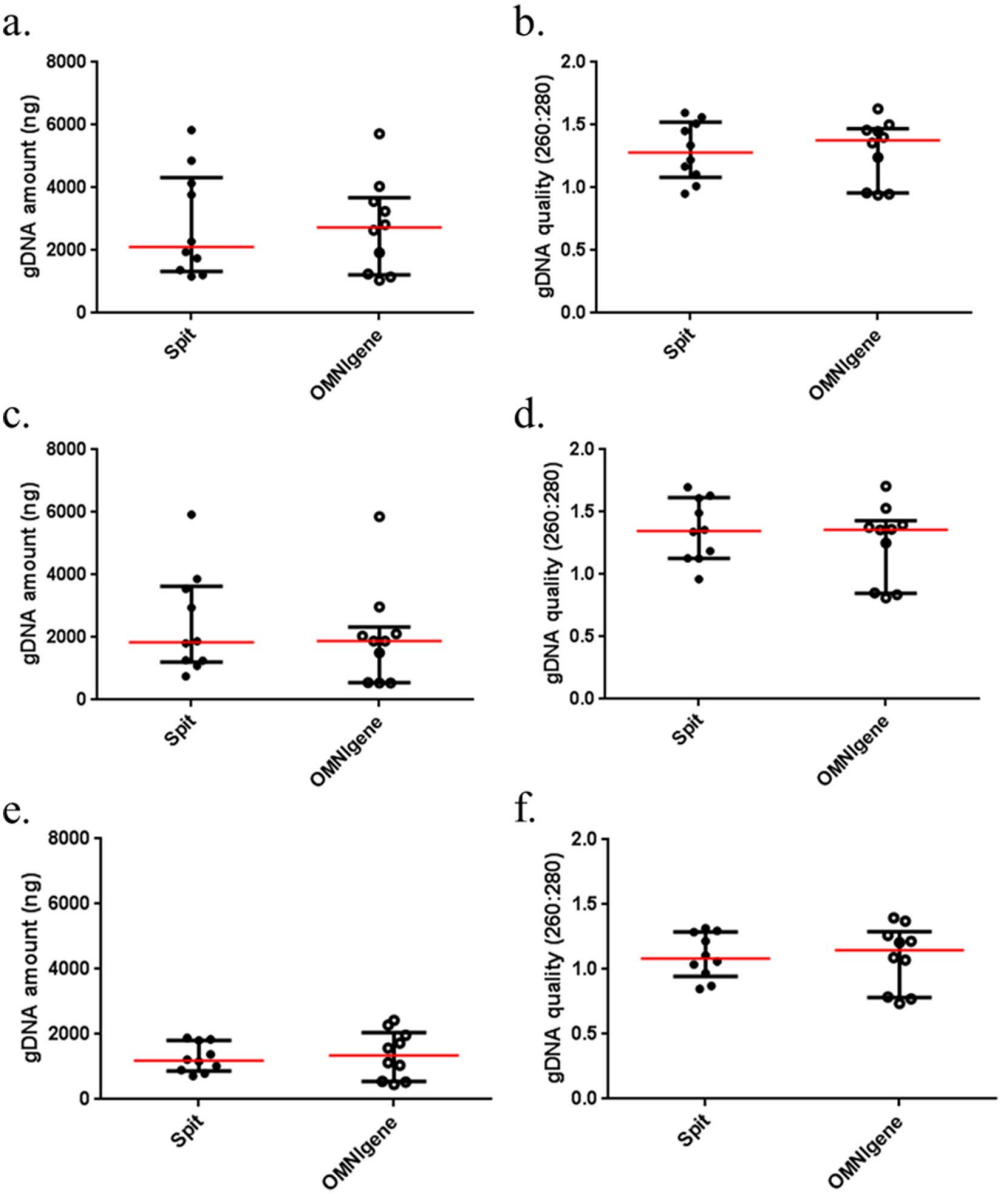
Extracted salivary gDNA from different saliva collection methods. Scatter plots for the average quantity and quality of the extracted gDNA triplicates from each collection and extraction method ﻿(a., b. Maxwell® 16 LEV blood DNA kit; c., d. in-house phenol-chloroform extraction and e., f. QIAamp DNA Microbiome Kit).

**Figure 3:**
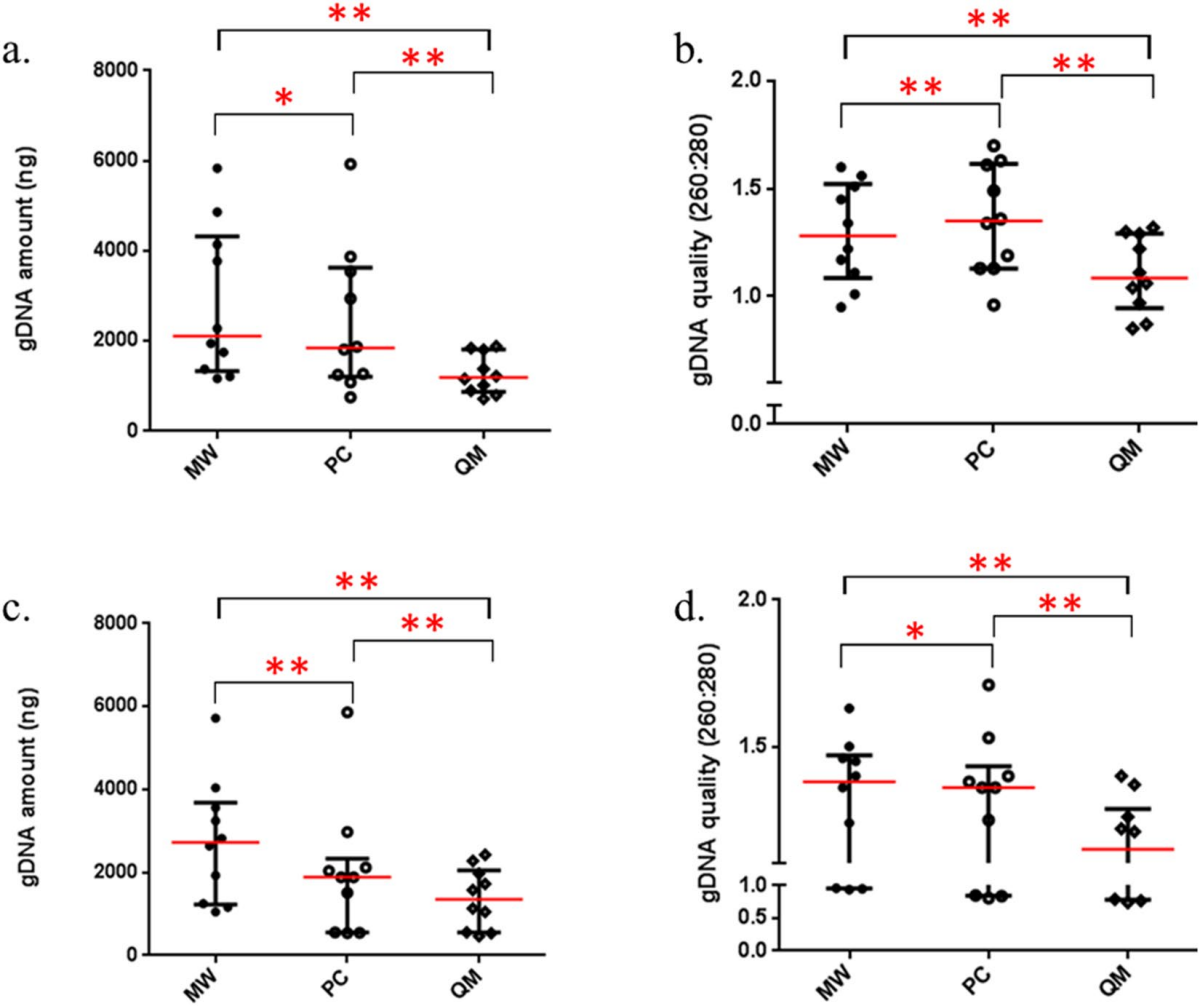
Extracted salivary gDNA from different bacterial gDNA extraction methods. Scatter plots for the average quantity and quality of the extracted gDNA triplicates from each collection (a., b. spit from 50 mL Falcon tube and c., d. OMNIgene) and extraction (MW represents Maxwell® 16 LEV blood DNA kit; PC represents in-house phenol-chloroform extraction and QM represents QIAamp DNA Microbiome Kit) method. Significant differences are denoted with *=p < 0.05, **p = < 0.01, ***=p < 0.001, ****=p < 0.0001 respectively.

There were no significant differences in the quantity and quality of extracted gDNA in saliva samples collected from the first normal healthy control cohort (n = 10) (Fig. 1a), demonstrating the resilient of salivary gDNA without the help of additional preservation system (Fig. 2). The salivary gDNA extraction was performed in triplicate and the results were consistent and reproducible (Supplementary Data 2). In contrast, the salivary gDNA quantity and quality significantly differed among the three extraction methods (Fig. 3). Irrespective of the collection method, the average gDNA quantity was high in spit samples using the Maxwell® 16 LEV blood DNA kit followed by the phenol-chloroform extraction method compared with a commercial QIAamp DNA Microbiome Kit method (Supplementary Data 3). However, the data were less variable when gDNA was extracted using the QIAamp DNA Microbiome Kit. In terms of gDNA quality, the 260/280 ratio of samples extracted with Maxwell® 16 LEV blood DNA kit were closer to the 1.8 ‘pure’ gDNA value. To ensure that the extracted gDNA is not contaminated with RNA, randomly selected gDNA samples were analysed on a 1% agarose gel electrophoresis. No RNA traces were detected by this analysis.

### Purity of isolated bacterial gDNA from various saliva fractions

Since there were no significant differences in the quantity and quality of gDNA extracted from the triplicate extraction as described above for each sample, gDNA was pooled and used as template for qPCR assays to determine the ratio of bacterial to human gDNA content. The qPCR assays’ efficiency were optimised using six-point serial dilution from 1.56 to 50 ng for the bacterial 16S rRNA gene and the human *β-globin* gene. Based on the results, the qPCR showed an average PCR efficiency of 0.99 for the bacterial 16S rRNA gene and an average efficiency of 0.98 for the human *β-globin* gene (Supplementary Data 4).

All spit samples were screened under the optimised condition. According to the results, Maxwell® 16 LEV blood DNA kit appeared to be a better extraction method with spit sample collected in 50 mL sterile Falcon tubes. The average ratio of threshold cycle (Ct) mean between extracted bacterial to human gDNA from spit samples collected in 50 mL Falcon tube using Maxwell® 16 LEV blood DNA kit was highest (Ct, 16.48:28.35 respectively) with multiple undetectable Ct means for the human *β-globin* gene (Table 1). It was noted that although the human *β-globin* gene was mostly undetected in extracted gDNA from spit samples collected in OMNIgene using the Maxwell® 16 LEV blood DNA kit, the average Ct mean for the bacterial 16S rRNA gene was significantly higher at 22.51, indicating a low abundance of bacterial gDNA (Table 1).

**Table 1:** Bacterial 16S rRNA and human *β-globin* qPCR statistical summary for the extracted gDNA (for all three extraction methods).

Sample	Spit		OMNIgene	
	Bacterial 16S rRNA/Ct mean	Human *β-globin*/Ct mean	Bacterial 16S rRNA/Ct mean	Human *β-globin/*Ct mean
**Maxwell® 16 LEV blood DNA kit**				
Average	16.48	28.35	22.51	29.81
Standard deviation	5.07	1.60	5.65	0.60
Standard error	1.60	0.51	1.79	0.19
**In-house phenol-chloroform extraction**				
Average	12.80	26.39	14.22	26.43
Standard deviation	2.63	2.44	6.12	2.83
Standard error	0.83	0.77	1.94	0.89
**QIAamp DNA Microbiome Kit**				
Average	14.83	27.47	15.89	27.26
Standard deviation	4.28	1.56	8.13	2.61
Standard error	1.35	0.49	2.57	0.83

Based on these collective results, the Maxwell® 16 LEV blood DNA kit appears to provide a more enriched bacterial gDNA extraction from our samples. The average Ct mean for the bacterial 16S rRNA gene from each extraction method did not significantly differ from one another while Maxwell® 16 LEV blood DNA kit was able to significantly reduce the content of human gDNA (Table 1). We also chose to collect samples in 50 mL sterile Falcon tubes and store at −80 °C for the second part of this study, in our hands, this collection method was more compatible with our subsequent use of the Maxwell® 16 LEV blood DNA kit.

### The influence of saliva fraction on microbial analysis

**Figure 4:**
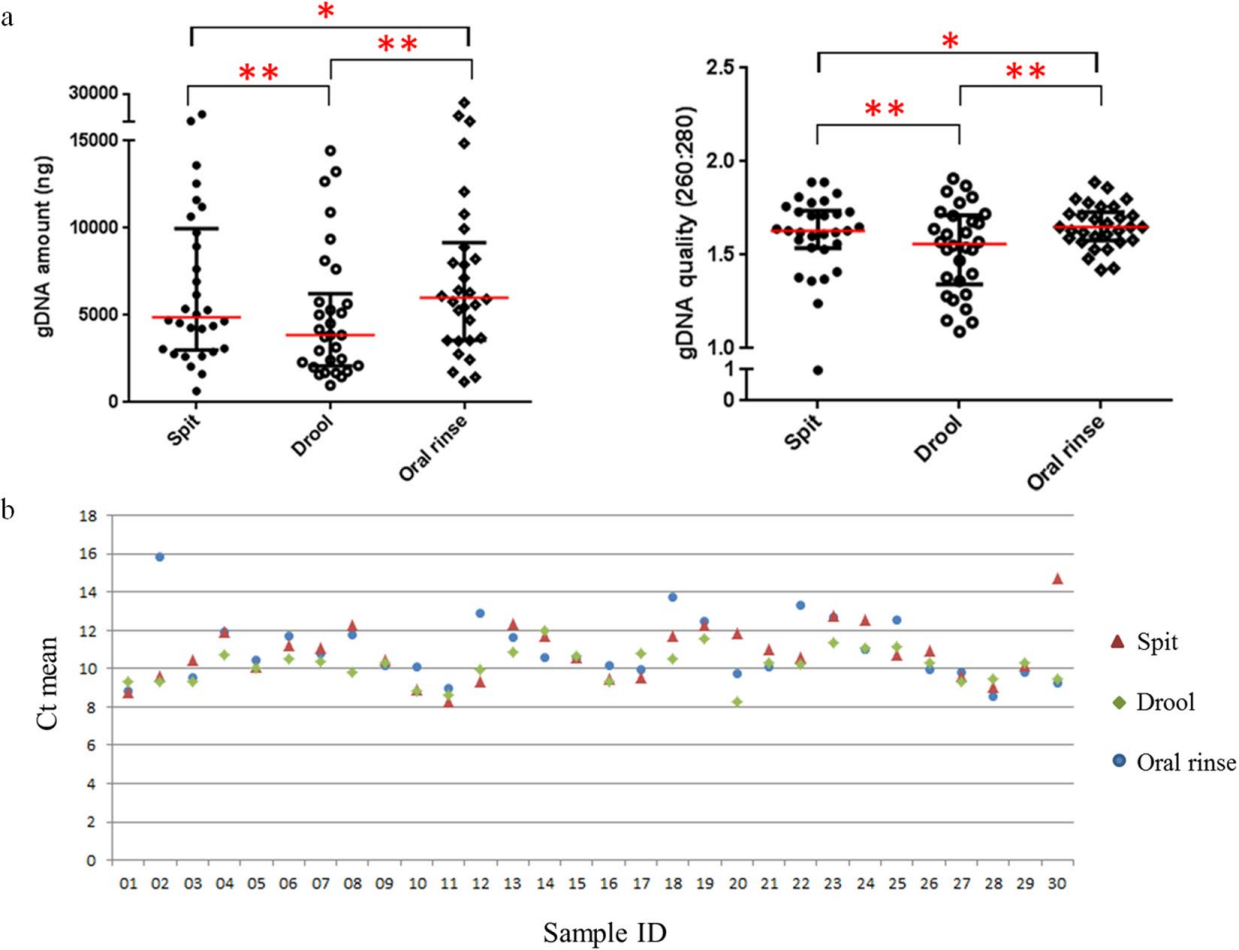
Extracted salivary gDNA from different saliva fractions. (**a**) Scatter plots for the quantity and quality of the extracted bacterial gDNA from each saliva fraction. Significant differences are denoted with *=p < 0.05, **=p < 0.01, ***=p < 0.001, ****=p < 0.0001 respectively. (**b**) Bacterial 16S rRNA and human *β-globin* qPCR Ct means distribution trend for the extracted gDNA from spit, drool and oral rinse.

From the second normal healthy control cohort (n = 30) (Fig. 1b), the quantity and quality of extracted gDNA were significantly different between saliva fractions (Fig. 4a). The purity of gDNA from the saliva fractions was determined using the 16S rRNA gene and the human *β-globin* gene qPCR assays as previously described. There were no significant differences between the purity of bacterial gDNA extracted from spit, drool and oral rinse (Fig. 4b).

Extracted gDNA from spit, drool and oral rinse samples from 10 randomly selected healthy individuals were subjected to 16S rRNA gene amplicon sequencing to compare the microbial diversity among different saliva fractions. Based on our data, the average length of reads is 500 bp and the OTU were subsampled to 5833 counts. After processing, 651760 high quality sequences were obtained in this study, with an average of 20651 sequences per sample. From these sequences, 6 known phyla and 30 genera were identified, and a total of 90 OTUs were detected at the 97% sequence identity threshold.

**Figure 5:**
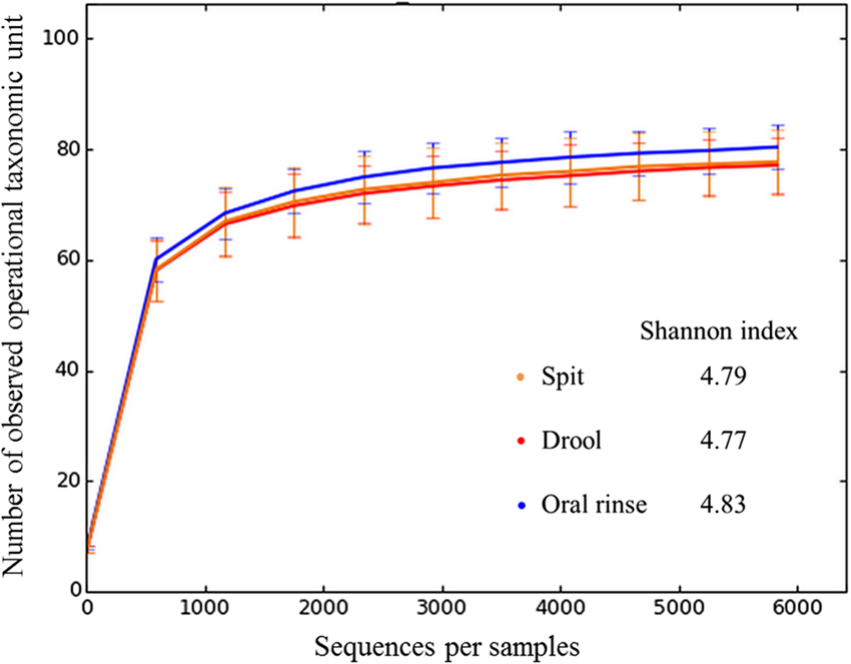
Rarefaction curve for observed operational taxonomic units against sequences per sample for spit, drool and oral rinse.

**Figure 6:**
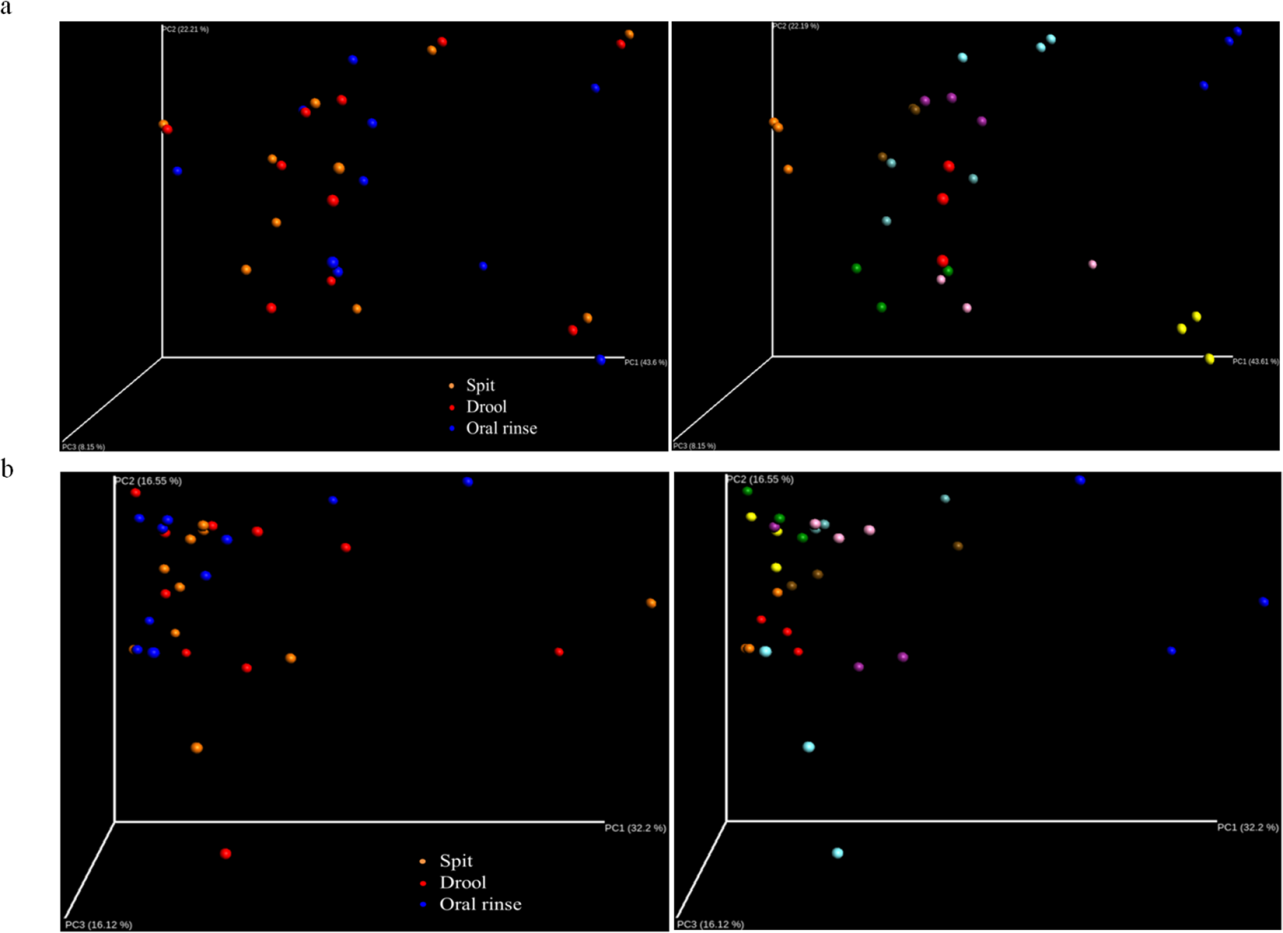
Beta-diversity of salivary microbiome. Weighted (**a**) and unweighted (**b**) PCoA plot for spit, drool and oral rinse samples with respective adjacent plots emphasizing on the subjects in different representing colours.

A rarefaction curve of the observed OTUs against sequences per sample was plotted for spit, drool and oral rinse to determine the efficiency of the sequencing process (Fig. 5). The Shannon index was determined for each sample, with the oral rinse samples having a consistently greater mean value (4.83) compared to spit and drool samples (4.79 and 4.77, respectively), although these differences were not statistically significant (Fig. 5). The β-diversity analyses showed no observable patterns among the spit, drool and oral rinse samples, the differences thereby primarily driven by the inter-subject variation rather than saliva fractions (Fig. 6).

**Figure 7:**
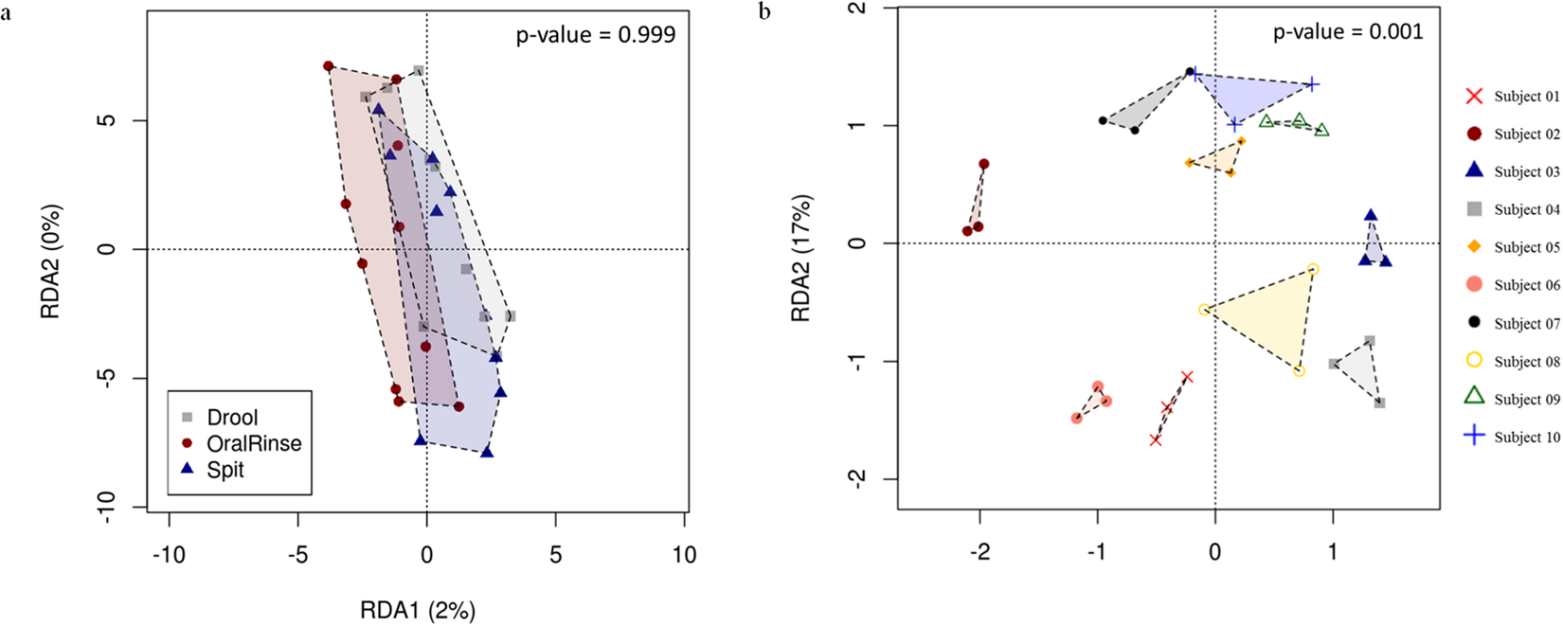
Salivary microbiome genera redundancy analysis on different (**a**) saliva fractions and (**b**) subjects.

The taxonomic profiles of spit, drool and oral rinse samples were examined based on the proportion of bacterial sequences determined at genus level (Supplementary Data 5). The five major genera found in all three saliva fractions were *Streptococcus* (17.5%), *Prevotella* (15.5%), *Veillonella* (15.3%), *Neisseria* (12.7%) and *Haemophilus* (10%). Calypso’s redundancy analysis was used to determine if combining the distribution pattern of all the genera from each saliva fraction into a panel could distinguish different saliva fractions (Fig. 7a). The p-value generated was 0.999, indicating that there are no significant differences. A second redundancy analysis under the same set of parameters was also carried out to investigate if the genera distribution of the three saliva fractions is distinguishable from one another (Fig. 7b). The results showed that the genera distributions from the three saliva fractions were significantly different for each individual with a p-value of 0.001, indicating the microbiome were more driven by host/environmental variations between subjects.

## Discussion

Saliva harbours bacteria shed from adhering microbial communities on various intraoral surfaces^26^. It is also well established that the oral cavity is colonized by numerous and diverse microorganisms^27^. While bacterial gDNA extraction methods have been rigidly investigated in the past, there is a paucity of data and knowledge regarding the ideal saliva fraction to be used for microbial analysis of the oral cavity^28–30^. According to Lazarevic *et al*.^28^, mechanical lysis (repeated bead-beating) is critical for greater bacterial OTU richness in saliva samples due to the rigid bacterial cell walls. This was also confirmed by Sohrabi *et al*.^29^ in addition to the incorporation of enzymatic cell lysis (repeated bead-beating in lysis buffer) to increase the purity of extracted bacterial gDNA. In this study, we incorporated the enzymatic-mechanical lysis method with current bacterial gDNA extraction kits to investigate the yield and quality of bacterial gDNA from different saliva fractions.

Our results demonstrate comparable quality and quantity of extracted gDNA from saliva samples when samples are either preserved using a stabilising buffer solution (OMNIgene) or when stored neat at −80 °C. In our hands, the Maxwell® 16 LEV Blood DNA kit with enzymatic-mechanical lysis provides the preferred method for isolating bacterial gDNA from saliva, in terms of gDNA yield, quality and ratio of bacterial:human gDNA. We have found also relatively higher α-diversity of OTUs in the oral rinse fraction as opposed to spit and drool, although these differences were not statistically significant.

Bacterial colonisation is apparent at many sites within the oral cavity, including the gingival, cervical line, tooth surface, tonsil, buccal mucosa, and tongue. As such it is important to develop a method that can access all of these sites and the diversity inherent to them^27, 31, 32^. Since microbes in the oral cavity travel via tongue and saliva movement, three saliva fractions were tested for microbial diversity via 16S rRNA gene amplicon sequencing^31, 33^. The main difference among these saliva fractions is that the muscle movements from spitting action may provide a larger microbial coverage compared with passive drooling; while oral rinse sampling provides access into the microbial populations residing within the oropharyngeal/throat area through the gargling action of the saline solution. The taxonomic profiles of the three saliva fractions were similar at genus-level and therefore can arguably be used inter-changeably for down-stream applications. However, we recommend the oral rinse method as the saliva fraction of choice when possible for future studies due to a greater α-diversity in OTUs compared to drool and split considering the inclusion of oropharyngeal regions. Furthermore, it should also be pointed out that healthy volunteers were used in this study, but in our experience an oral rinse sample is ideal in people restricted in their ability to produce saliva, such as patients with xerostomia or cancer patients who have undergone chemoradiation treatment.

The bacterial profiles identified in our study corroborated with previous findings. Bik *et al*.^34^ investigated the oral microbiome of ten individuals with healthy oral tissues and gingiva (full-mouth clinical examinations were performed by certified dentists) in both men and women between the ages of 27 to 61 years^34^. Whole-mouth saliva samples and dental plaque specimens were collected and pooled to create a single ‘subgingival pool’ in this study^34^. Similarly, Ozga *et al*.^35^ examined the oral microbiome of 57 individuals (no selection criteria) from Oklahoma, United States^35^. Whole-mouth saliva samples were collected from both men and women between the ages of 23 to 70 years^35^. The oral microbiome profiles from both of these studies were determined by 16S rRNA gene amplicon sequencing^34, 35^. The result from our study bears a closer resemblance with findings from Bik *et al*.^34^ probably due to the similarly stringent recruitment criteria used in subject selection^34^. In contrast, Ozga *et al*.^35^ selected more lenient recruitment criteria that included subjects with smoking history, bad oral hygiene and active antibiotic use^35^. The microbial profiles produced from all three saliva fractions were comparable with the numerically predominant taxa included in both studies despite minor differences in abundance, indicating the reliability of our dataset.

## Conclusion

Saliva sample collection, processing and gDNA preparation does not significantly influence the salivary microbiome profiles provided that enzymatic-mechanical lysis has been incorporated into the bacterial gDNA extraction protocol. Based on our findings, saliva sampling for microbial research is fairly flexible compared to faecal samples. This allows for number of modifications based on the experimental set-up and down-stream application. However, to improve the purity of bacterial gDNA extracted from saliva samples, we found the Maxwell® 16 LEV Blood DNA kit provided the optimal results. The lack of a coherent and comprehensive guideline in salivary microbiome study design might be a major driver for conflicting findings, a limit inter-study comparison derived from the published literature. We hope that our findings will contribute to those efforts to standardise the saliva collection and bacterial gDNA extraction protocol for future microbiome research.

## Declarations

### Ethics

This study is approved by the University of Queensland’s (HREC no. 2014000679 and 2017000662) and Queensland University of Technology’s (HREC no. 1400000617) Medical Ethical Institutional Board and the Princess Alexandra Hospital’s (PAH) Ethics Review Board (HREC no. HREC/12/QPAH/381). All participants were informed consent for this study.

### Availability of data and materials

All data and materials supporting the findings of this study can be found in the body of the manuscript as well as in the supplementary section.

## Electronic supplementary material

Supplementary Dataset 1

## References

[1] na Head and Neck Cancer Biomarkers Detected in Saliva. *Cancer Biol Ther* **4**, 6–12 (2005).

[2] Pepe MS (2001). Phases of Biomarker Development for Early Detection of Cancer. J Natl Cancer Inst.

[3] Lucs AV, Saltman B, Chung CH, Steinberg BM, Schwartz DL (2013). Opportunities and challenges facing biomarker development for personalized head and neck cancer treatment. Head Neck.

[4] Bandhakavi S, Stone MD, Onsongo G, Van Riper SK, Griffin TJ (2009). A dynamic range compression and three-dimensional peptide fractionation analysis platform expands proteome coverage and the diagnostic potential of whole saliva. J. Proteome Res..

[5] Iorgulescu G (2009). Saliva between normal and pathological. Important factors in determining systemic and oral health. J Med Life.

[6] Pfaffe T, Cooper-White J, Beyerlein P, Kostner K, Punyadeera C (2011). Diagnostic potential of saliva: current state and future applications. Clin Chem.

[7] Malamud D (2011). Saliva as a diagnostic fluid. Dent Clin North Am.

[8] Chai RC (2016). A pilot study to compare the detection of HPV-16 biomarkers in salivary oral rinses with tumour p16INK4a expression in head and neck squamous cell carcinoma patients. BMC Cancer.

[9] Lim Y (2016). Salivary DNA methylation panel to diagnose HPV-positive and HPV-negative head and neck cancers. BMC Cancer.

[10] Ovchinnikov DA (2014). DNA Methylation at the Novel CpG Sites in the Promoter of MED15/PCQAP Gene as a Biomarker for Head and Neck Cancers. Biomark Insights.

[11] Ovchinnikov DA (2012). Tumor-suppressor Gene Promoter Hypermethylation in Saliva of Head and Neck Cancer Patients. Transl Oncol.

[12] Salazar C (2014). A novel saliva-based microRNA biomarker panel to detect head and neck cancers. Cell Oncol (Dordr).

[13] Zhang, X. *et al*. Quantification of D-dimer levels in human saliva. *Bioanalysis* **5** (2013). 10.4155/bio.13.190 24053240

[14] Zhang X, Dimeski G, Punyadeera C (2014). Validation of an immunoassay to measure plasminogen-activator inhibitor-1 concentrations in human saliva. Biochem Med.

[15] Wong DT (2015). Salivary extracellular noncoding RNA: emerging biomarkers for molecular diagnostics. Clin Ther.

[16] Nunes LA, Mussavira S, Bindhu OS (2015). Clinical and diagnostic utility of saliva as a non-invasive diagnostic fluid: a systematic review. Biochem Med (Zagreb).

[17] Guerrero-Preston, R. *et al*. 16S rRNA amplicon sequencing identifies microbiota associated with oral cancer, Human Papilloma Virus infection and surgical treatment. *Oncotarget* (2016). 10.18632/oncotarget.9710 PMC5239478 27259999

[18] Weidlich P, Cimões R, Pannuti CM, Oppermann RV (2008). Association between periodontal diseases and systemic diseases. Braz Oral Res.

[19] Shacter, E. & Weitzman, S. A. Chronic inflammation and cancer. *Oncology (Williston Park)* **16**, 217–226, 229; discussion 230–212 (2002). 11866137

[20] Vogelmann R, Amieva MR (2007). The role of bacterial pathogens in cancer. Curr Opin Microbiol.

[21] Chen M (2016). The impact of different DNA extraction methods on the analysis of microbial diversity of oral saliva from healthy youths by polymerase chain reaction-denaturing gradient gel electrophoresis. JDS.

[22] Jenkinson HF (2011). Beyond the oral microbiome. Environ Microbiol.

[23] Shanahan ER, Zhong L, Talley NJ, Morrison M, Holtmann G (2016). Characterisation of the gastrointestinal mucosa-associated microbiota: a novel technique to prevent cross-contamination during endoscopic procedures. Aliment Pharmacol Ther.

[24] Haas BJ (2011). Chimeric 16S rRNA sequence formation and detection in Sanger and 454-pyrosequenced PCR amplicons. Genome Res.

[25] McDonald D (2012). An improved Greengenes taxonomy with explicit ranks for ecological and evolutionary analyses of bacteria and archaea. ISME J.

[26] Takeshita T (2016). Bacterial diversity in saliva and oral health-related conditions: the Hisayama Study. Sci Rep.

[27] Dewhirst FE (2010). The human oral microbiome. J Bacteriol.

[28] Lazarevic V, Gaia N, Girard M, Francois P, Schrenzel J (2013). Comparison of DNA extraction methods in analysis of salivary bacterial communities. PLoS One.

[29] Sohrabi M (2016). The yield and quality of cellular and bacterial DNA extracts from human oral rinse samples are variably affected by the cell lysis methodology. J Microbiol Methods.

[30] Vesty A, Biswas K, Taylor MW, Gear K, Douglas RG (2017). Evaluating the Impact of DNA Extraction Method on the Representation of Human Oral Bacterial and Fungal Communities. PLoS ONE.

[31] Hull MW, Chow AW (2007). Indigenous microflora and innate immunity of the head and neck. Infect Dis Clin North Am.

[32] Taylan I (2011). Comparison of the Surface and Core Bacteria in Tonsillar and Adenoid Tissue With Beta-Lactamase Production. Indian J Otolaryngol Head Neck Surg.

[33] Danser MM, Gómez SM, Van der Weijden GA (2003). Tongue coating and tongue brushing: a literature review. Int J Dent Hyg.

[34] Bik EM (2010). Bacterial diversity in the oral cavity of ten healthy individuals. The ISME J.

[35] Ozga, A.T. *et al*. Oral microbiome diversity among Cheyenne and Arapaho individuals from Oklahoma. *Am J Phys Anthropol* (2016). 10.1002/ajpa.23033 27357925

