# Supplementary material for: The saliva microbiome profiles are minimally affected by collection method or DNA extraction protocols
Source: Sci Rep. 2017 Aug 17;7:8523. doi: 10.1038/s41598-017-07885-3 (PMC5561025; doi:10.1038/s41598-017-07885-3)
Supplement: Supplementary file 1 — Supplementary Dataset 1 [file 41598_2017_7885_MOESM1_ESM.docx]

**The saliva microbiome profiles are minimally affected by collection method or DNA extraction protocols (supplementary data).**

Yenkai Lim^1,2^, Makrina Totsika^1^, Mark Morrison^3^ and Chamindie Punyadeera^1,2*^

^(1)^The School of Biomedical Sciences, Institute of Health and Biomedical Innovation, Queensland University of Technology, 60 Musk Avenue, Kelvin Grove, Brisbane, QLD 4059, Australia, ^(2)^Translational Research Institute, Woolloongabba, Brisbane, QLD, 4102, Australia, ^(3)^The University of Queensland Diamantina Institute, The University of Queensland, Translational Research Institute, Woolloongabba, Brisbane, QLD, 4102, Australia.

*Correspondence:

Associate Professor Chamindie Punyadeera

The School of Biomedical Sciences

Institute of Health and Biomedical Innovations, Queensland University of Technology

60 Musk Avenue, GPO Box 2434

Brisbane QLD 4059

Australia.

+61 7 3138 0830

| Name | Email |
| --- | --- |
| Yenkai LIM | [y43.lim@qut.edu.au](mailto:y43.lim@qut.edu.au) |
| Makrina TOTSIKA | [makrina.totsika@qut.edu.au](mailto:makrina.totsika@qut.edu.au) |
| Mark MORRISON | m.morrison1@uq.edu.au |
| Chamindie PUNYADEERA | [chamindie.punyadeera@qut.edu.au](mailto:chamindie.punyadeera@qut.edu.au) |

Supplementary data 1.Subject recruitment criteria and demography

1. Must have a good general state of health and aged between 20 and 30 years.
2. No fever or signs and symptoms suggesting active infection/illness on the day of saliva donation.
3. No mouth ulcers, inflammation of the gums, halitosis, any mouth infection, dry mouth or sensitive teeth on the day of intended donation of saliva.
4. No history of Hepatitis A or B.
5. No ear-nose-throat complaints.
6. Not undergoing dental treatment
7. Not wearing dentures.
8. No history of diabetes or hypoglycaemia.
9. No history of allergies including food allergies.
10. Not pregnant, planning to become pregnant in the near future or breast-feeding.
11. Not on medication such as lipid-lowering drugs, hormonal-replacement therapy, and evidence of hepatic dysfunction or supplements other than contraceptives.
12. No recent history of alcohol or drug abuse or other medical condition.
13. No prior history of any cancer. Participants with family history of cancer however, can be included.
14. No previous irradiation to head and neck region.

| Parameter | Aim 1 | Aim 2 |
| --- | --- | --- |
| Number of participant | 20 | 30 |
| Age | 24 (23-28) | 26 (23-30) |
| Gender (M:F) | 12:8 | 19:11 |

Supplementary data 2. Bacterial gDNA extraction reproducibility test


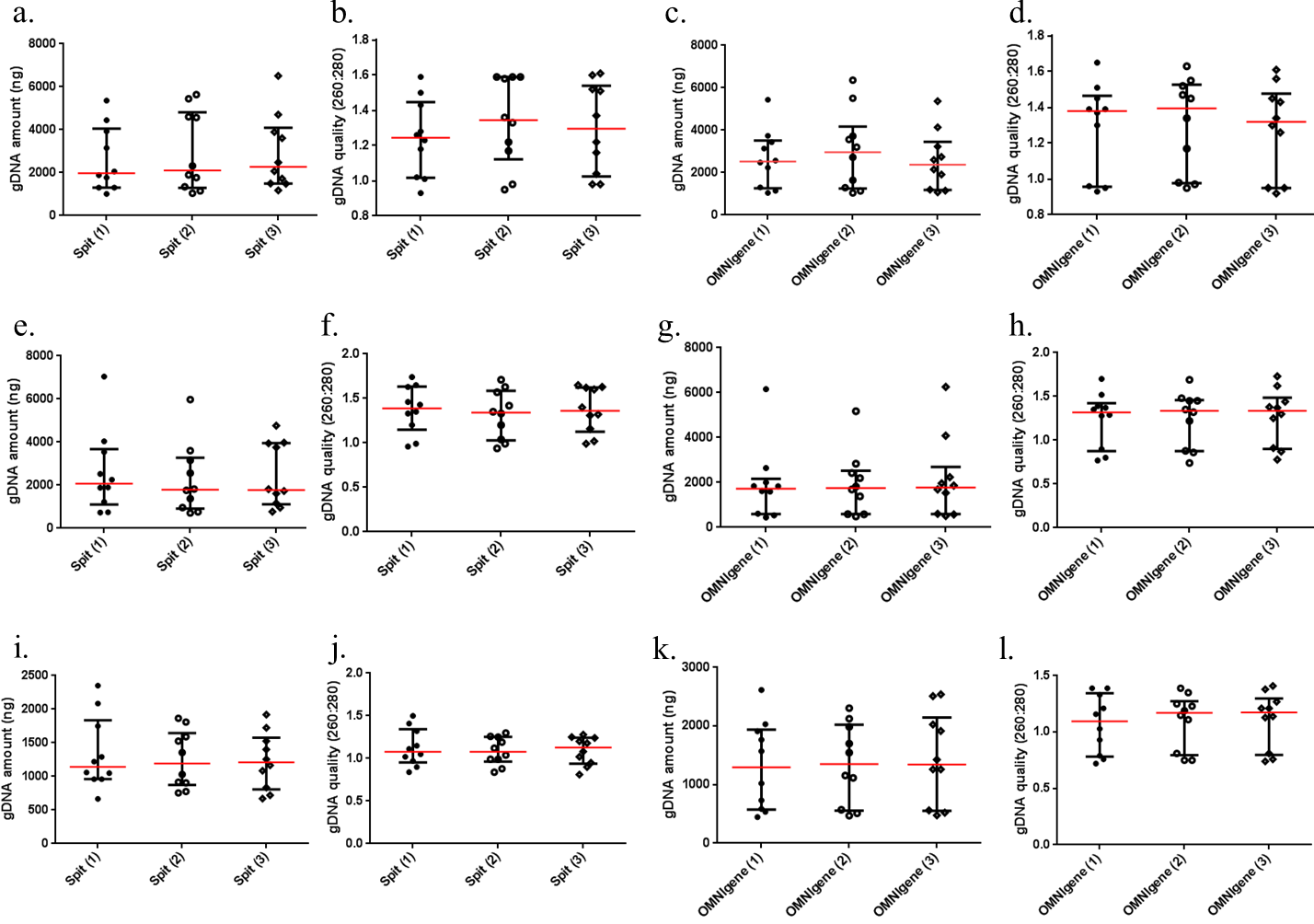


Scatter plots for the quantity and quality of the extracted gDNA replicates from each collection and extraction method (a., b., c., d. Maxwell® 16 LEV blood DNA kit; e., f., g., h. in-house phenol-chloroform extraction and i., j., k., l. QIAamp DNA Microbiome Kit).

Supplementary data 3. Statistical summary of extracted salivary gDNA from different saliva collection methods.

|  | Spit (gDNA amount, ng) | | | Spit (gDNA quality, 260:280) | | |
| --- | --- | --- | --- | --- | --- | --- |
|  |  |  |  |  |  |  |
|  | MW (n=30) | PC  (n=30) | QM  (n=30) | MW | PC | QM |
| Average | 2769.43 | 2435.07 | 1273.83 | 1.29 | 1.35 | 1.10 |
| Standard deviation | 405.41 | 418.86 | 156.96 | 0.24 | 0.26 | 0.18 |
| Standard error | 234.06 | 241.83 | 90.62 | 0.04 | 0.04 | 0.04 |
|  |  |  |  |  |  |  |
|  | OMNIgene (gDNA amount/ng) | | | OMNIgene (gDNA quality/260:280) | | |
|  |  |  |  |  |  |  |
|  | MW  (n=30) | PC  (n=30) | QM  (n=30) | MW | PC | QM |
| Average | 2742.78 | 1992.27 | 1339.17 | 1.30 | 1.25 | 1.09 |
| Standard deviation | 405.03 | 334.19 | 158.58 | 0.26 | 0.30 | 0.24 |
| Standard error | 233.85 | 192.94 | 91.56 | 0.03 | 0.03 | 0.02 |
|  |  |  |  |  |  |  |

MW, PC and QM represent Maxwell® 16 LEV blood DNA kit, in-house phenol-chloroform extraction and QIAamp DNA Microbiome Kit respectively.


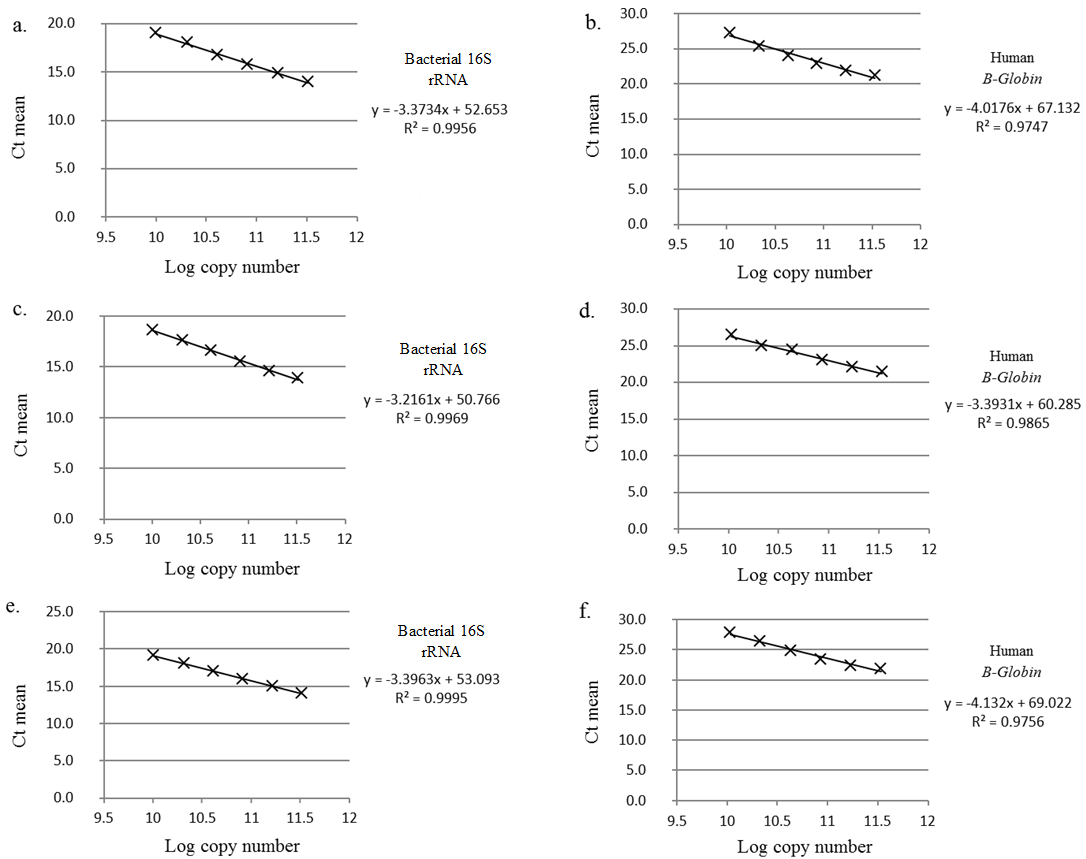
Supplementary data 4. qPCR optimisation

qPCR efficiencies for the bacterial 16S rRNA gene (1114F; 1221R) and the human *β-globin* gene for spit samples extracted by Maxwell® 16 LEV blood DNA kit (a. and b.), in-house phenol-chloroform extraction (c. and d.) and QIAamp DNA Microbiome Kit (e. and f.) with their respective R^2^ values (Ct represents threshold cycle).

Supplementary data 5. Salivary microbiome taxonomy summary


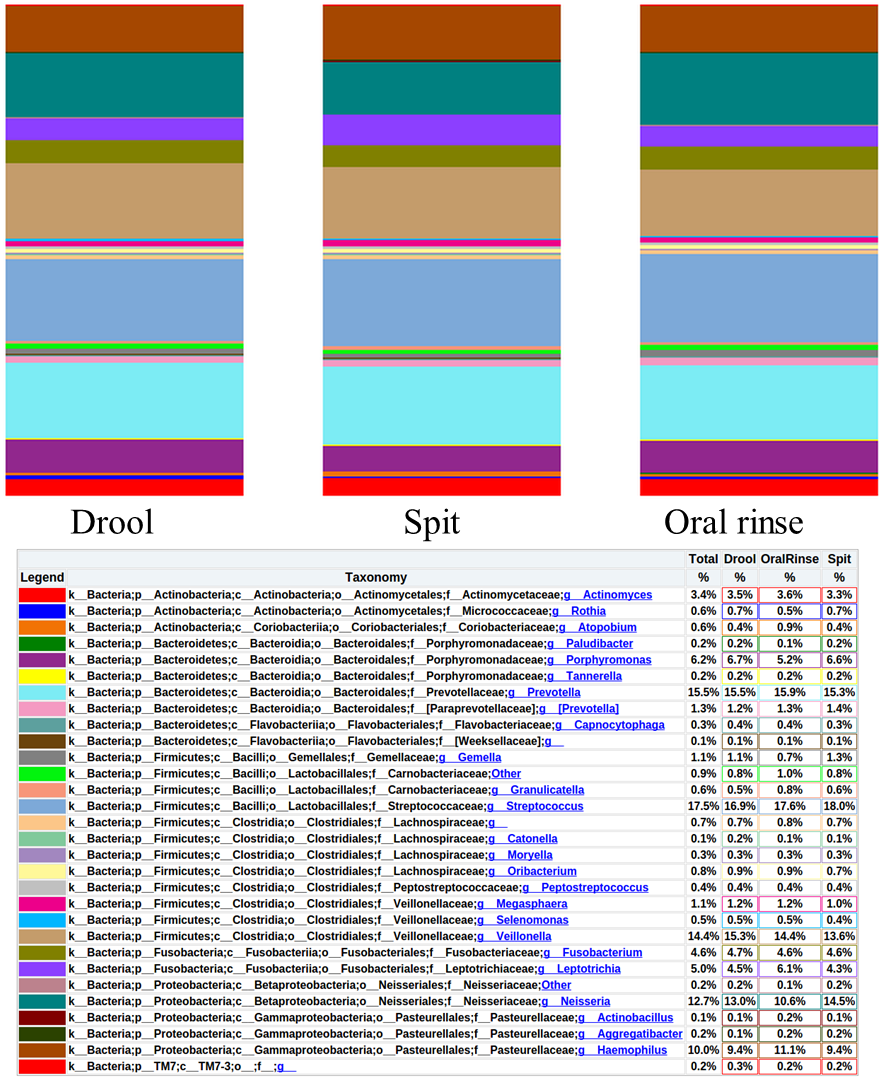


Taxonomy bar chart based on the proportion of bacteria sequences in spit, drool and oral rinse at genus-level.

Supplementary data 6. Salivary microbiome genera distribution of proportion statistical comparison for different saliva fractions (spit, drool and oral rinse).

| Taxa | P | P.Bonf | FDR qValue | OralRinse.mean | Drool.mean | Spit.median | OralRinse.median | Spit.mean | Drool.median |
| --- | --- | --- | --- | --- | --- | --- | --- | --- | --- |
| Rothia | 0.18 | 1.00 | 0.98 | 0.004542 | 0.007195 | 0.004885 | 0.003340 | 0.007033 | 0.0059100 |
| Granulicatella | 0.26 | 1.00 | 0.98 | 0.007629 | 0.005295 | 0.005830 | 0.008145 | 0.005762 | 0.0043700 |
| Atopobium | 0.26 | 1.00 | 0.98 | 0.008618 | 0.004212 | 0.003085 | 0.006170 | 0.003718 | 0.0030850 |
| Aggregatibacter | 0.32 | 1.00 | 0.98 | 0.002224 | 0.001338 | 0.000000 | 0.000256 | 0.001865 | 0.0000000 |
| Actinobacillus | 0.47 | 1.00 | 0.98 | 0.001715 | 0.001418 | 0.000000 | 0.000515 | 0.001290 | 0.0000850 |
| Gemella | 0.48 | 1.00 | 0.98 | 0.007019 | 0.011414 | 0.007200 | 0.005910 | 0.013101 | 0.0078000 |
| Catonella | 0.49 | 1.00 | 0.98 | 0.001405 | 0.001697 | 0.001030 | 0.001545 | 0.001269 | 0.0015400 |
| Porphyromonas | 0.66 | 1.00 | 0.98 | 0.052030 | 0.067389 | 0.075800 | 0.061150 | 0.066167 | 0.0644000 |
| Neisseria | 0.66 | 1.00 | 0.98 | 0.105909 | 0.129754 | 0.149000 | 0.085900 | 0.145239 | 0.1160000 |
| Carnobacteriaceae_Other | 0.67 | 1.00 | 0.98 | 0.009923 | 0.008353 | 0.006940 | 0.012400 | 0.008271 | 0.0078850 |
| Oribacterium | 0.69 | 1.00 | 0.98 | 0.008745 | 0.008544 | 0.006260 | 0.007200 | 0.007108 | 0.0066850 |
| X.Prevotella | 0.75 | 1.00 | 0.98 | 0.013103 | 0.012122 | 0.012350 | 0.006860 | 0.013956 | 0.0093300 |
| TM7.3_o__f__g__ | 0.76 | 1.00 | 0.98 | 0.001799 | 0.002519 | 0.001715 | 0.001370 | 0.002315 | 0.0016300 |
| Paludibacter | 0.76 | 1.00 | 0.98 | 0.001216 | 0.001868 | 0.000425 | 0.001030 | 0.002365 | 0.0004250 |
| Prevotella | 0.77 | 1.00 | 0.98 | 0.158630 | 0.154650 | 0.121000 | 0.154000 | 0.152530 | 0.1270000 |
| Selenomonas | 0.78 | 1.00 | 0.98 | 0.004813 | 0.005173 | 0.001970 | 0.002740 | 0.003667 | 0.0030850 |
| Tannerella | 0.79 | 1.00 | 0.98 | 0.002125 | 0.001838 | 0.000600 | 0.000600 | 0.001970 | 0.0003400 |
| Actinomyces | 0.81 | 1.00 | 0.98 | 0.035701 | 0.034501 | 0.030000 | 0.030850 | 0.033130 | 0.0301500 |
| Megasphaera | 0.81 | 1.00 | 0.98 | 0.012256 | 0.011548 | 0.005485 | 0.009595 | 0.010159 | 0.0082300 |
| Veillonella | 0.81 | 1.00 | 0.98 | 0.143880 | 0.152910 | 0.119000 | 0.156500 | 0.135560 | 0.1645000 |
| Moryella | 0.88 | 1.00 | 0.98 | 0.003290 | 0.003460 | 0.002055 | 0.002400 | 0.002742 | 0.0021450 |
| Leptotrichia | 0.89 | 1.00 | 0.98 | 0.060978 | 0.045260 | 0.024400 | 0.032050 | 0.042933 | 0.0255500 |
| Neisseriaceae_Other | 0.92 | 1.00 | 0.98 | 0.001353 | 0.002040 | 0.000770 | 0.000255 | 0.002125 | 0.0007700 |
| Peptostreptococcus | 0.92 | 1.00 | 0.98 | 0.004268 | 0.004306 | 0.002740 | 0.003945 | 0.003977 | 0.0030000 |
| X.Weeksellaceae._g__ | 0.92 | 1.00 | 0.98 | 0.001302 | 0.001440 | 0.001370 | 0.001285 | 0.001388 | 0.0015400 |
| Fusobacterium | 0.93 | 1.00 | 0.98 | 0.045810 | 0.046497 | 0.038350 | 0.035550 | 0.046126 | 0.0353000 |
| Capnocytophaga | 0.93 | 1.00 | 0.98 | 0.003666 | 0.003672 | 0.000860 | 0.001630 | 0.002757 | 0.0014600 |
| Haemophilus | 0.94 | 1.00 | 0.98 | 0.111450 | 0.093880 | 0.091250 | 0.104500 | 0.094020 | 0.0912000 |
| Streptococcus | 0.95 | 1.00 | 0.98 | 0.176360 | 0.168690 | 0.160000 | 0.200000 | 0.180120 | 0.1550000 |
| Lachnospiraceae_g__ | 0.98 | 1.00 | 0.98 | 0.007975 | 0.006757 | 0.005055 | 0.004800 | 0.007313 | 0.0054000 |

End.
